# Association between OX40L polymorphism and type 2 diabetes mellitus in Iranians

**DOI:** 10.1186/s12920-024-01958-9

**Published:** 2024-07-09

**Authors:** Abdolreza Sotoodeh Jahromi, Saiedeh Erfanian, Abazar Roustazadeh

**Affiliations:** 1 Research Center for Noncommunicable Diseases, Jahrom University of Medical Sciences, Jahrom, Iran; 2 Immunology Department, School of Medicine, Jahrom University of Medical Sciences, Jahrom, Iran; 3 Department of Biochemistry, School of Medicine, Jahrom University of Medical Sciences, Jahrom, Iran; 4 Department of Advanced Medical Sciences and Technologies, School of Medicine, Jahrom University of Medical Sciences, Jahrom, Iran

**Keywords:** OX40L, Polymorphism, Diabetes mellitus, Middle East

## Abstract

**Introduction:**

Diabetes mellitus (DM) is one of the leading causes of morbidity and mortality worldwide. It is a multifactorial disease that genetic and environmental factors contribute to its development. The aim of the study was to investigate the association of OX40L promoter gene polymorphisms with type 2 diabetes mellitus (T2DM) in Iranians.

**Materials and methods:**

Three hundred and sixty-eight subjects including 184 healthy subjects and 184 T2DM patients were enrolled in our study. Polymerase chain reaction-restriction fragment length polymorphism (PCR-RFLP) was applied to detect genotype and allele frequencies of rs3850641, rs1234313 and rs10912580. In addition, SNPStats web tool was applied to estimate haplotype frequency and linkage disequilibrium (LD).

**Results:**

The distribution of tested polymorphisms was statistically different between the T2DM patients and healthy subjects (*P* < 0.01). rs1234313 AG (OR = 0.375, 95% CI = 0.193–0.727, *P* = 0.004) and rs10912580 AG (OR = 0.351, 95% CI = 0.162–0.758, *P* = 0.008) genotypes were associated with the decreased risk of T2DM in Iranians. Moreover, our prediction revealed that AAG (OR = 0.46, 95% CI= (0.28–0.76), *P* = 0.0028) and GAG (OR = 0.24, 95% CI= (0.13–0.45), *P* < 0.0001) haplotypes were related to the reduced risk of the disease. However, the tested polymorphisms had no effect on biochemical parameters and body mass index (BMI) in the patient group (*P* > 0.05).

**Conclusion:**

Our findings revealed that OX40L promoter gene polymorphisms are associated with T2DM. Moreover, genotype and allelic variations were related to the decreased risk of T2DM in Iranians. Further studies are recommended to show whether these polymorphic variations could affect OX40/OX40L interaction or OX40L phenotype.

**Supplementary Information:**

The online version contains supplementary material available at 10.1186/s12920-024-01958-9.

## Introduction

Diabetes mellitus (DM) is one of the leading causes of morbidity and mortality worldwide, that all members of society are exposed to it. Global burden of disease reports in 2021 indicated that there were 529 million peoples living with DM and 96% of them suffered from type 2 diabetes mellitus (T2DM) [1]. Hyperglycemia, which is the main symptom of T2DM, may be due to decreased secretion or resistance of peripheral tissues to insulin [2, 3] So far, a definitive treatment for diabetes has not been discovered; however, the available treatments include improving the quality of life, reducing weight and treating obesity, or using drugs such as metformin [4, 5].

T2DM is known as a chronic, low grade inflammatory disease [6]. Studies indicated that apparent changes in the activity of the immune system occur in diabetic patients, especially in adipose tissue, pancreatic islets, liver, vasculature and circulating leukocytes, and inflammation plays a pivotal role in the pathogenesis of T2DM and its complications [7]. Adipose tissues are one of the most important targets of insulin and dysfunction of these tissues could affect insulin function. White adipose tissue (WAT), especially visceral WAT suggested to be one of the most important sources of inflammatory markers in T2DM and it is a major target of inflammation process in patients suffering from diabetes [8]. WAT is a source of inflammatory markers or substances involved in the inflammatory pathways, such as tumor necrosis factor-Alpha (TNF-α), interleukin (IL) -1, IL-6, chemokines, adipokines and other substances [9–11]. In addition, inflammation in beta cells of Langerhans islets leads to the reduction of beta cells number and function [12].

OX40L (Gene ID: 7292; https://www.ncbi.nlm.nih.gov/gene/7292) which is also known as cluster of differentiation (CD) 252, tumor necrosis factor super family-4 (TNFSF4), CD134L and glycoprotein34 (GP34) is a member of TNF superfamily. [13]. It is a glycoprotein contained 156 amino acids with a cytoplasmic tail (23 amino acids) and an extracellular domain (133 amino acids). It is the ligand of OX40 and mainly expressed on antigen presenting cells (APCs), such as B cells, dendritic cells and macrophages [14]. The expression of OX40L is upregulated in response to antigen presentation on APCs in the islets of Langerhans [15, 16]. OX40/OX40L pair has an important role in second signaling pathways in T cells [14, 17]. Studies have shown that IL-18, interferon-gamma (IFN -γ), and transmission of inflammatory signals through toll-like receptors could induce OX40L expression on APCs [18, 19]. Pro-inflammatory factors including CD28 ligation, CD40L ligation and IFN-γ induce the expression of OX40 and OX40L. Interaction of OX40 and OX40L promote T cell survival and lead to an effector T cell phenotype. Expression of OX40L may also induce by activated CD4^+^and CD8^+^ T cells [14]. In vitro studies indicated that neutralizing of OX40L in OX40 + cells isolated from a mouse model could inhibit the production of IFN -γ and TNF-α [20, 21]. Previous studies have shown that OX40/OX40L signal pathway is important in autoimmune diseases such as rheumatoid arthritis, Graves’ hyperthyroidism and systemic lupus erythematosus (SLE) [22, 23]. Some studies have suggested that T2DM is an autoimmune disease that involves both innate and adaptive immunity [24, 25]. Therefore, inflammation is considered to have a critical role in developing T2DM and its complications. It has been reported that the enhancement of OX40/OX40L signal pathway promotes the proliferation, activation of fat-infiltrating T lymphocyte and insulin resistance in obese mice, while, the inflammation and immune response of adipose tissue in OX40 knockout mice is reduced [7]. Therefore, the OX40/OX40L signaling pathway plays an important role in the development of insulin resistance in T2DM [26]. Some studies indicated that blocking OX40/OX40L interactions could suppress diabetes progression [27, 28]. However, the contribution of OX40L, especially gene polymorphisms, in developing of T2DM needs to be studied.

OX40L gene polymorphisms have been studied in SLE [29], bladder cancer [30], cerebral arterial thrombosis [31], and atherosclerotic disorders [32]. The aim of the study was to investigate the association of rs3850641, rs1234313 and rs10912580 polymorphisms in the promoter region of OX40L gene with T2DM in Iranian patients.

## Materials and methods

### Subjects

Three hundred and sixty eight subjects including 184 healthy subjects (aged 26–88) and 184 T2DM patients (aged 35–84) referred to Peymanieh hospital (Jahrom city, Iran) were enrolled in the study (from January 2019 to February 2020). The number of patients referring to the endocrinology department of the hospital for follow-up of diabetes varies depending on different times and was around 4000–5000 patients per year. It is noted that most of the patients are those who went to the endocrinologist for a check-up or follow-up treatment, and except for routine tests, no money has been received from the patients for specialized tests, and the cost has been provided by the research plan. Healthy subjects were the staffs working in Jahrom University of Medical Sciences. The minor allele frequency (MAF) of rs3850641, rs1234313 and rs10912580 was 0.16, 0.31 and 0.21, respectively. Hence, the sample size was estimated based on the MAF of rs3850641 which had the rarest frequency according to the data reported by dbSNP database (https://www.ncbi.nlm.nih.gov/snp/). Diabetic patients had fasting plasma glucose (FPG) ≥ 126 mg/dl [1]. Patients with underlying diseases (cancer, liver, autoimmune diseases, primary hyperlipidemia, cardiovascular and kidney disorders), infectious, pregnant individuals and diabetic patients undergoing dialysis were excluded from the study. Except for the new cases of T2DM, other patients used injectable drugs (Insulin and/or glucagon-like peptide-1 receptor agonist) and/or oral drugs (metformin, sulfonylureas, thiazolidindiones, inhibitors of dipeptidyl peptidase 4 (DPP4i/DPP-4 or gliptins) and sodium-glucose co-transporter-2(SGLT2) inhibitors). FPG, high density lipoprotein cholesterol (HDL-C), low density lipoprotein cholesterol (LDL-C), total cholesterol (TC) and triacylglycerol (TG) were measured by Biorex company kits (Catalog numbers for FPG, TG, TC, HDL-C and LDL-C kits were BXC0101, BXC0271, BXC0261, BXC0420 and BXC0430B, respectively). Body mass index (BMI) calculated by kg/m^2^. All participants filled out a written consent form.

### Polymerase chain reaction-restriction fragment length polymorphism (PCR-RFLP)

Whole blood samples were taken from the subjects. Genomic DNA was extracted by salting out method and immediately stored at -80 ˚C [33]. In this study rs3850641, rs1234313 and rs10912580 in the promoter region of OX40L gene were investigated. The sequences of primers are summarized in supplementary Table 1 [34].

PCR reactions were performed in a final volume of 20 µl containing 10 µl Taq DNA Polymerase Master Mix Red (AMPLIQON, Cat no: A180301), 2.5 µl genomic DNA (0.2 µg), 1 µl forward primer (1µM), 1 µl reverse primer (1µM) and 5.5 µl PCR grade water.

PCR conditions were an initial denaturation at 94 ˚C for 5 min followed by 35 cycles of 94 ˚C for 30 s, 55 ˚C for 30 s, 72 ˚C for 1 min and a final extension at 72 ˚C for 10 min.

Then, PCR products were applied to RFLP. The rs3850641 PCR product (274 bp) was digested by *HpyCH4III* (Fermentas Company, 10 U, 16 h overnight) to detect G/G (210 and 64 bp), A/G (210, 64 and 274 bp) and A/A (274 bp) genotypes. Moreover, the rs1234313 PCR product (305 bp) was digested by *BsrDI* (Fermentas Company, 10 U, 16 h overnight) to detect A/A (165 and 140 bp), A/G (165,140 and 305 bp) and G/G (305 bp) genotypes. The rs10912580 PCR product (372 bp) was digested by *AccI* (Fermentas Company, 10 U, 16 h overnight) to detect G/G (199 and 173 bp), A/G (199,173 and 372 bp) and A/A (372 bp) genotypes. Digested products were run on 3% agarose gel and visualized by the green viewer on a UV transilluminator.

### Statistical analysis

SPSS v.18 (Chicago) was used for statistical analysis. Normality of the data was checked by Kolmogorov-smirnov test. Hardy-Weinberg equilibrium (HWE) was performed to survey allele deviation. Odds ratio (OR) obtained by binary logistic regression. The numeric data were reported as mean ± standard error (SE). The differences in FPG, TG, HDL-C, LDL-C, TC and age between the healthy group and T2DM patients were analyzed by student t test. In addition, the differences of genotype, allele and sex frequencies between the healthy subjects and patients were analyzed by chi square test. Also, chi square test was applied to investigate the differences of allele and genotype distributions in the men and woman of the both groups. Two-way multivariate analysis of variance (Two-way MANOVA) was performed to investigate the effect of independent variables (Polymorphisms) on dependent variables (Biochemical parameters such as FBG, TC, TG, HDL-C and LDL-C, and BMI). Wilks lambda and tukey tests were used in Two-way MANOVA. *A p* value less than 0.05 was considered to be significant. Haplotype frequencies estimation and linkage disequilibrium analysis were performed by SNPStats web tool [35]. Frequency threshold for rare haplotypes was 0.01. Gene interactions were investigated under four different models (codominant, dominant, recessive modes and overdominant).

## Results

### Characteristics of the study population

Demographic and biochemical findings of our study population are summarized in Table 1. Our study recruited 368 subjects including 184 healthy controls and 184 T2DM patients. The distribution of men and women in both the groups was not significantly different (*P* > 0.5). However, the mean age of the patients was statistically higher than the controls (*P* < 0.001). All the biochemical laboratory findings, except for the HDL, including FBG, TG, TC and HDL-C were statistically different between the healthy subjects and T2DM patients (*P* < 0.001). The BMI index in the patient group was significantly higher than that of the control group (*P* < 0.001).

**Table 1:** Characteristics of the study population

Demographic and Biochemical Parameters	Control(*n* = 184)	T2DM (*n* = 184)	*P* value
**Age** (year)	51.91 ± 0.77	55.9 ± 0.8	< 0.001
**BMI** (kg/m^2^)	26.16 ± 0.25	27.94 ± 0.32	< 0.001
**FBG** (mg/dL)	92.21 ± 0.66	207.71 ± 3.94	< 0.001
**TC** (mg/dL)	167.58 ± 2.08	194.59 ± 2.74	< 0.001
**HDL-C** (mg/dL)	42.69 ± 0.75	46.27 ± 1.87	0.078
**LDL-C** (mg/dL)	94.71 ± 1.53	120.78 ± 2.95	< 0.001
**TG** (mg/dL)	141.83 ± 3.72	186.31 ± 5.09	< 0.001
**Sex**(male/female)	50/134	54/130	0.72

Data are reported as Mean ± SE except for sex. BMI: body mass index, FBG: fasting blood glucose, HDL-C: high density lipoprotein cholesterol, LDL-C: low density lipoprotein cholesterol, SE: standard error, TC: total cholesterol, T2DM: type 2 diabetes mellitus, TG: triacylglycerol. The differences in demographic and biochemical parameters were analyzed by student t and chi square tests

### Genotype and allele distributions

#### rs3850641 genotype and allele frequencies

Table 2 summarized the genotype and allele distributions of the tested polymorphisms in T2DM and healthy subjects. rs3850641 A and G allele frequencies were statistically different between the groups (*P* < 0.001). The results revealed that G allele was associated with the decreased risk of T2DM in Iranians (OR = 0.487, 95% CI = 0.336–0.706, *P* < 0.001). Also, rs3850641 A/A, A/G and G/G genotypes distribution was different between T2DM and control groups (*P* < 0.001). However, there was no relation between the distribution of genotypes and risk of the disease (*P* > 0.05). In addition, distribution of A/G + G/G versus A/A was higher in the patients (*P* < 0.001) and A/G + G/G decreased the risk of T2DM (OR = 0.415, 95% CI = 0.266–0.645, *P* < 0.001).

**Table 2:** Genotype and allele distribution of OX40L gene in the control and T2DM subjects

Allele/Genotype		Controls (*n* = 184)	T2DM (*n* = 184)	*P* value	OR (CI 95%)
**rs3850641**					
Allele	A	314(85%)	272(74%)	< 0.001	0.487(0.336–0.706) ^**a**^
	G	54(15%)	96(26%)		
Genotype	G/G	8(4.3%)	14(7.6%)	< 0.001	1.023(0.393–2.657)
	A/G	38(20.7%)	68(37%)		0.422(0.171–1.045)
	A/A	138(75%)	102(55.4%)		Ref
	A/G + G/G	46(25%)	82(44.6%)	< 0.001	0.415(0.266–0.645) ^a^
**rs1234313**					
Allele	G	253(68.8%)	204(55.4%)	< 0.001	0.565(0.418–0.764) ^a^
	A	115(31.2%)	164(44.6%)		
Genotype	G/G	89(48.4%)	50(27.2%)	< 0.001	Ref
	A/G	75(40.8%)	104(56.5%)		0.375(0.193–0.727) ^b^
	A/A	20(10.9%)	30(16.3%)		0.924(0.488–1.751)
	A/G + A/A	95(51.6%)	134(72.8%)	< 0.001	0.398 (0.258–0.615) ^a^
**rs10912580**					
Allele	A	242(65.5%)	206(56%)	0.008	0.662(0.492–0.892) ^c^
	G	126(34.5%)	162(44%)		
Genotype	G/G	13(7.1%)	24(13%)	0.009	0.618(0.299–1.277)
	A/G	100(54.3%)	114(62%)		0.351(0.162–0.758) ^d^
	A/A	71(38.6%)	46(25%)		Ref
	A/G + G/G	113(61.4%)	138(75%)	0.007	0.531(0.339–0.829) ^e^

^**a**^*P* value was < 0.001. ^**b**^*P* value was 0.004.^**c**^*P* value was 0.007.^**d**^*P* value was 0.008.^**e**^*P* value was 0.005. OR: odds ratio, Ref: reference genotype or allele. The differences in allele and genotype distributions were calculated by chi-square test. Odds ratio was obtained by binary logistic regression

#### rs1234313 genotype and allele frequencies

The distribution of rs1234313 genotype and allele frequencies was statistically different between the groups (*P* < 0.001). Our analysis indicated that allelic variation (OR = 0.565, 95% CI = 0.418–0.764, *P* < 0.001) and A/G genotype (OR = 0.375, 95% CI = 0.193–0.727, *P* = 0.004) are linked to the reduced risk of T2DM. However, the results showed that there is no relation between A/A genotype and the risk of the disease (*P* > 0.05). Moreover, the distribution of A/G + A/A versus G/G was statistically different between the groups and A/G + G/G decreased the risk of the disease (*P* < 0.001).

#### rs10912580 genotype and allele frequencies

Frequencies of rs10912580 A and G alleles were different between the controls and T2DM patients (*P* = 0.008). Our results indicated that G allele was associated with the low risk of the disease (OR = 0.662, 95% CI = 0.492–0.892, *P* = 0.007). Moreover, rs10912580 A/G and G/G genotypes were different between the groups (*P* = 0.009). The results showed that A/G (OR = 0.351, 95% CI = 0.162–0.758, *P* = 0.008) genotype was associated with the decreased risk of T2DM. Frequency of A/G + G/G versus A/A was higher in the patients (*P* < 0.007) and A/G + G/G decreased the risk of the disease (OR = 0.531, 95% CI = 0.339–0.829, *P* < 0.005).

### Frequencies of the tested polymorphisms between the men and women

The distribution of the tested polymorphisms was not significantly different between women and men (*P* > 0.05). Also, the genotype and allele distributions of the polymorphisms were the same among women and men with T2DM (*P* > 0.0.05).

### HWE analysis

HWE results for the tested polymorphisms are presented in supplementary Table 2. rs3850641 and rs1234313 in the patient group were in accordance with HWE. In addition, rs1234313 in population study was in accordance with HWE. However, rs3850641 and rs10912580 in all the subjects were not in accordance with HWE.

### Univariate and multivariate analysis of variance

Univariate analysis of variance showed that rs3850641in population study has a significant relation to FPG (*P* = 0.02). Analysis revealed that FPG in A/G genotypes (163.75 ± 6.68) is significantly higher than that of A/A genotypes (142.61 ± 4.44, *P* = 0.025). This result was not confirmed by Two-way MANOVA (Wilks’ Lambda > 0.05). However, univariate analysis of variance showed that the tested polymorphisms had no effect on biochemical parameters in the patient group (*P* > 0.05). In addition, Two-way MANOVA showed that there is a relation between rs1234313 and FPG in population study. Analysis revealed that FPG is significantly different between G/G (130.180 ± 5.752) and A/G (160.950 ± 5.068, *P* < 0.001) and also between G/G and A/A (165.640 ± 9.590, *P* = 0.005) in the population study (Wilks’ Lambda = 0.019). However, Two-way MANOVA revealed that the tested polymorphisms had no effect on biochemical parameters and BMI in T2DM patients.

### Polymorphism-to-polymorphism interaction and T2DM

Polymorphic site interactions are summarized in Tables 3, 4 and 5. Analysis assumed that G allele of rs3850641, A allele of rs1234313, and G allele of 10,912,580 are the risk alleles. As showed in Table 3, predicted models of rs3850641 indicated that A/G in the codominant model (*P* = 4e-04), A/G-G/G in the dominant model (1e-04) and A/G in the overdominant model (5e-04) are related to the decreased risk of T2DM. In addition, predicted models of rs1234313 (Table 4) revealed that A/G and AA in the codominant model (*P* = 1e-04), A/G-A/A in the dominant model(*P* < 0.0001) and A/G in the overdominant model (*P* = 0.0024) were in association with the decreased risk of T2DM. Predicted models of 10,912,580 are summarized in Table 5. Analysis showed that A/G and G/G in the codominant model (*P* = 0.0081), and A/G-G/G in the dominant model (*P* = 0.005) are related to the low risk in the patient group.

**Table 3:** rs3850641 association with response status (*n* = 368, crude analysis)

Model	Genotype	Status T2DM	Status Control	OR (95% CI)	*P* value		
**Codominant**	A/A	102 (55.4%)	138 (75%)	1.00	4e-04		
	A/G	68 (37%)	38 (20.6%)	**0.41 (0.26–0.66)**			
	G/G	14 (7.6%)	8 (4.3%)	0.42 (0.17–1.04)			
**Dominant**	A/A	102 (55.4%)	138 (75%)	1.00	1e-04		
	A/G-G/G	82 (44.6%)	46 (25%)	**0.41 (0.27–0.65)**			
**Recessive**	A/A-A/G	170 (92.4%)	176 (95.7%)	1.00	0.18		
	G/G	14 (7.6%)	8 (4.3%)	0.55 (0.23–1.35)			
**Overdominant**	A/A-G/G	116 (63%)	146 (79.3%)	1.00	5e-04		
	A/G	68 (37%)	38 (20.6%)	**0.44 (0.28–0.71)**			

OR: odds ratio. Polymorphic site interactions were calculated by SNPStats web tool

**Table 4:** rs1234313 association with response status (*n* = 368, crude analysis)

Model	Genotype	status = Ca	status = Co	OR (95% CI)	*P*-value
**Codominant**	G/G	50 (27.2%)	89 (48.4%)	1.00	1e-04
	A/G	104 (56.5%)	75 (40.8%)	**0.41 (0.26–0.64)**	
	A/A	30 (16.3%)	20 (10.9%)	**0.37 (0.19–0.73)**	
**Dominant**	G/G	50 (27.2%)	89 (48.4%)	1.00	< 0.0001
	A/G-A/A	134 (72.8%)	95 (51.6%)	**0.40 (0.26–0.62)**	
**Recessive**	G/G-A/G	154 (83.7%)	164 (89.1%)	1.00	0.13
	A/A	30 (16.3%)	20 (10.9%)	0.63 (0.34–1.15)	
**Overdominant**	G/G-A/A	80 (43.5%)	109 (59.2%)	1.00	0.0024
	A/G	104 (56.5%)	75 (40.8%)	**0.53 (0.35–0.80)**	

OR: odds ratio. Polymorphic site interactions were calculated by SNPStats web tool

**Table 5:** rs10912580 association with response status (*n* = 368, crude analysis)

Model	Genotype	Status T2DM	Status Control	OR (95% CI)	*P*-value
**Codominant**	A/A	46 (25%)	71 (38.6%)	1.00	0.0081
	A/G	114 (62%)	100 (54.4%)	**0.57 (0.36–0.90)**	
	G/G	24 (13%)	13 (7.1%)	**0.35 (0.16–0.76)**	
**Dominant**	A/A	46 (25%)	71 (38.6%)	1.00	0.005
	A/G-G/G	138 (75%)	113 (61.4%)	**0.53 (0.34–0.83)**	
**Recessive**	A/A-A/G	160 (87%)	171 (92.9%)	1.00	0.055
	G/G	24 (13%)	13 (7.1%)	0.51 (0.25–1.03)	
**Overdominant**	A/A-G/G	70 (38%)	84 (45.6%)	1.00	0.14
	A/G	114 (62%)	100 (54.4%)	0.73 (0.48–1.11)	

OR: odds ratio. Polymorphic site interactions were calculated by SNPStats web tool

Linkage balance effect of three polymorphic sites of OX40L gene are summarized in supplementary Table 3. Multiple-SNP analysis to calculate linkage disequilibrium showed that rs3850641- rs1234313 (D^’^=0.7591, *r* = 0.4915, *P* < 0.001), rs3850641-10912580(D^’^=0.2651, *r* = 0.1673, *P* < 0.001) and rs1234313-10912580(D^’^=0.4368, *r* = 0.4256, *P* < 0.001) are in linkage disequilibrium.

### Haplotype distribution

Haplotype frequency estimation is summarized in supplementary Table 4. AGA was the most frequent haplotype in T2DM (0.4106); however, the frequency was lower than that of the control group (0.5025). GGA was the lowest haplotype observed in studied groups. The association between haplotypes and T2DM is shown in Table 6. Our prediction revealed that AAG (OR = 0.46, 95% CI= (0.28–0.76), *P* = 0.0028) and GAG (OR = 0.24, 95% CI= (0.13–0.45), *P* < 0.0001) haplotypes were in association with the reduced risk of T2DM. Association between haplotype frequency and gender was investigated (Supplementary Table 5). Our results showed that AGA had a protective role in males, whilst AAG and GAG had a protective role in both the genders.

**Table 6:** Haplotype association with response (*n* = 368, crude analysis)

	snp1	snp2	snp3	Freq	OR (95% CI)	*P*-value		
1	A	G	A	0.4578	1.00	---		
2	A	G	G	0.133	0.94 (0.56–1.58)	0.81		
3	A	A	G	0.131	**0.46 (0.28–0.76)**	0.0028		
4	G	A	G	0.1193	**0.24 (0.13–0.45)**	< 0.0001		
5	A	A	A	0.0744	0.85 (0.47–1.54)	0.59		
6	G	A	A	0.0544	0.72 (0.36–1.43)	0.35		
7	G	G	A	0.022	0.63 (0.19–2.07)	0.45		
rare	*	*	*	0.0081	0.39 (0.03–5.40)	0.48		
**Global haplotype association p-value: 0.00016**								

Freq: Frequency, OR: odds ratio, Snp1: rs3850641, Snp2: rs1234313, Snp3: rs10912580. Haplotype frequency estimations were performed by SNPStats web tool

## Discussion

The main finding of our study was that rs3850641, rs1234313 and rs10912580 in the promoter region of OX40L gene had a different distribution in T2DM patients compared to healthy subjects and the tested polymorphisms were associated with the decreased risk of T2DM. T2DM is the most common metabolic disorder worldwide. It is a multifactorial disease that genetic and environmental factors contribute to its development [3, 36].

Fu group investigated the association between rs3850641 polymorphism in OX40L gene and coronary heart disease (CHD) in an evidence based meta-analysis study [37]. They found that there is no association between this polymorphism and CHD. Contrary to the FU findings, our results showed that rs3850641 genotypes and alleles have a different distribution in T2DM compared to healthy subjects. In addition, G allele was associated with the low risk of T2DM in Iranians.

Our results revealed that the distribution of alleles and genotypes of rs1234313 was different between the groups. Moreover, A allele and A/G genotype were in relation to the decreased risk of the disease. Jiang group [31] investigated this polymorphism in the Han Chinese population with ischemic stroke. They showed that rs1234313 G/G + G/A versus A/A genotypes are correlated with large artery atherosclerosis (LAA) and small vessel disease (SVD). In contrast to our study, they have assumed the A allele as the reference allele. However, our finding indicated that G/A + A/A versus G/G decreased the risk of T2DM in Iranians.

Yuan group [34] studied rs1234313, rs3850641and rs10912580 in the northeast Chinese Han population with breast cancer. They claimed that rs3850641G allele could increase the susceptibility to breast cancer and rs10912580 A allele was associated with breast cancer. Our findings indicated that the tested polymorphisms were in association with T2DM. In contrast to Yuan, we found that rs10912580 G allele was in association with the decreased risk of the disease. The differences observed between the results of our study and other studies may be due to racial differences or differences in the studied diseases.

OX40L along with OX40 has an important role in the regulation of immune responses mediated by T cell. OX40/OX40L interaction is involved in the pathogenesis of autoimmune and inflammatory diseases [22, 23]. Zhang group findings indicated that the response of mucosal-associated invariant T (MAIT) cell alters in T2DM. Costimulatory TNF superfamily receptor OX40 is highly expressed in MAIT cells of these patients. In addition, OX40-positive MAIT cells show a high activation and a memory phenotype in comparison to OX40-negative MAIT cells. Moreover, OX40 expression have a negative correlation with the frequency of MAIT cells in the peripheral blood of T2DM patients [6]. However, our search in dbSNP, Google and PubMed databases showed no study that investigated the tested polymorphism in relation to DM especially in Iranian patients. Hence, this is the first study that investigated these polymorphisms in Iranian T2DM patients. Our results revealed that the tested polymorphisms were in association with the reduced risk of T2DM. Since these polymorphisms are located in promoter region of OX40L, it is worth studying whether the genotypes have an effect on the expression of OX40L and interaction of OX40/OX40L or not. An group investigated membrane (m) OX40 and mOX40L as well as the levels of soluble(s) OX40 and sOX40L in type 1 diabetes mellitus (T1DM). Their findings showed that sOX40 and sOX40L in the sera of T1DM patients were significantly higher than that of healthy subjects. They also claimed the down-regulation of mOX40 and mOX40L in T1DM patients which were correlated with the clinical characteristics and inflammatory factors [28].

Codominant, dominant and over dominant model of rs3850641, and codominant and dominant model of rs1234313 and rs10912580 were in association with the decreased risk of T2DM. In addition, the couples of rs3850641-rs1234313, rs3850641-rs10912580 and rs1234313-10912580 were in linkage disequilibrium. These results suggest that these polymorphic sites are probably inherited together in the Iranian population, or they are located in polymorphic sites where recombination has not occurred with a high percentage.

Haplotype frequencies estimation showed that AGA was the most frequent haplotype in the patients. Moreover, global haplotype association indicated that AAG and GAG haplotypes are associated with T2DM and these haplotypes decreased the risk of the disease in Iranians.

Biochemical parameters in the patient group were significantly higher than that of the control group. Since diabetic patients have a high tendency to develop complications related to diabetes such as cardiovascular diseases (CVD) and lipid markers has been proven to be a risk factor of CVD [38], therefore, we performed a multivariate analysis to observe if there is a relationship between the tested polymorphisms as independent factors and biochemical indices as dependent variables. MANOVA showed that there is a relation between rs1234313 and FPG in population study. However, the tested polymorphisms had no effect on biochemical parameters and BMI in T2DM patients.

### Limitation of the study

According to our knowledge, the current study was the first study that investigated OX40L polymorphisms in the Iranian T2DM patients. The sample size was relatively small; therefore it is suggested to use a larger sample size for future studies, especially in non-Iranian races, so that racial differences can be identified better. In the current study, indirect haplotyping technique was used, so it is necessary to use direct haplotyping technique in future studies to better study the genetic continuity and the effects of haplotypes on biochemical parameters.

## Conclusion

Our findings revealed that OX40L promoter gene polymorphisms are associated with T2DM. Moreover, rs3850641 G allele, and rs1234313 and rs10912580 A/G genotypes decreased the risk of T2DM in Iranian patients. Further studies are recommended to show that these polymorphic variations could affect OX40/OX40L interaction or OX40L phenotype. Our sample size was relatively small, so the findings should be interpreted with caution.

### Electronic supplementary material

Below is the link to the electronic supplementary material.

Supplementary Material 1

Supplementary Material 2

## Data Availability

The dataset generated and/or analyzed during the current study is available as supplementary Table 6. Also, data would be available upon a reasonable request from the corresponding author.

## Abbreviations

APCs: Antigen presenting cells
BMI: Body mass index
CD: cluster of differentiation
CHD: Coronary heart disease
DM: Diabetes mellitus
DPP4i/DPP-4: inhibitors of dipeptidyl peptidase 4
FPG: Fasting plasma glucose
GP34: glycoprotein34
HDL-C: High density lipoprotein cholesterol
HWE: Hardy-Weinberg equilibrium
IFN-γ: interferon-gamma
LAA: large artery atherosclerosis
IL: interleukin
LDL-C: Low density lipoprotein cholesterol
m: membrane
MAIT: mucosal-associated invariant T
PCR-RFLP: Polymerase chain reaction-restriction fragment length polymorphism
s: soluble
SGLT2: sodium-glucose co-transporter-2
SLE: Systemic lupus erythematosus
SE: Standard error
SVD: small vessel disease
TC: Total cholesterol
T1DM: type 1 diabetes mellitus
T2DM: Type 2 diabetes mellitus
TG: triacylglycerol
TNF-α: tumor necrosis factor-Alpha
TNFSF4: tumor necrosis factor super family-4
Two-way MANOVA: Two-way multivariate analysis of variance
WAT: White adipose tissue
