# Supplementary material for: Association between OX40L polymorphism and type 2 diabetes mellitus in Iranians
Source: BMC Med Genomics. 2024 Jul 9;17:184. doi: 10.1186/s12920-024-01958-9 (PMC11232195; doi:10.1186/s12920-024-01958-9)
Supplement: Supplementary file 1 — Supplementary Material 1 [file 12920_2024_1958_MOESM1_ESM.docx]

**Supplementary table 1** Sequences of primers used for detection of rs3850641, rs1234313 and 10912580 in OX40L gene

| Primers | Sequence |
| --- | --- |
| rs3850641.F | 5ʹ- GAACTGGTCTCTTTCCTATTTC -3ʹ |
| rs3850641.R | 5ʹ- CCCACAGCAATCGTAAAG-3ʹ |
| rs1234313.F | 5ʹ- CTCCTACCATGTCTCAAAC-3ʹ |
| rs1234313.R | 5ʹ- CTGTCTTCCACAGTCCTC-3ʹ |
| rs10912580.F | 5ʹ- CAGGAGGATCATTTGAACC-3ʹ |
| rs10912580.R | 5ʹ- CTTCGATGGAGACCATAAAC-3ʹ |

**Supplementary table 2** Hardy-Weinberg equilibrium calculation results for the tested- polymorphisms

| **Status** | **rs3850641** | **rs1234313** | **rs10912580** |
| --- | --- | --- | --- |
| Control | 0.032 | 0.49 | 0.0055 |
| T2DM | 0.5719 | 0.073 | 0.00059 |
| All subjects | 0.036 | 0.58 | <0.0001 |

Values are the P values of Hardy-Weinberg equilibrium calculation

**Supplementary Table 3** Multiple-SNP analysis to calculate linkage disequilibrium

| **D statistic** | Snp1 | Snp2 | Snp3 |
| --- | --- | --- | --- |
| Snp1 | . | 0.0961 | 0.0329 |
| Snp2 | . | . | 0.1008 |
| Snp3 | . | . | . |
| **D' statistic** | Snp1 | Snp2 | Snp3 |
| Snp1 | . | 0.7591 | 0.2651 |
| Snp2 | . | . | 0.4368 |
| Snp3 | . | . | . |
| **r statistic** | Snp1 | Snp2 | Snp3 |
| Snp1 | . | 0.4915 | 0.1673 |
| Snp2 | . | . | 0.4256 |
| Snp3 | . | . | . |
| **P-values** | Snp1 | Snp2 | Snp3 |
| Snp1 | . | 0 | 0 |
| Snp2 | . | . | 0 |
| Snp3 | . | . | . |

Snp1: rs3850641; Snp2: rs1234313; Snp3: rs10912580

**Supplementary Table 4** Haplotype frequencies estimation (n=368) in study population

|  | **snp1** | **snp2** | **snp3** | **Total** | **T2DM** | **Control** | **Cumulative frequency** |
| --- | --- | --- | --- | --- | --- | --- | --- |
| 1 | A | G | A | 0.4577 | 0.4106 | 0.5025 | 0.4577 |
| 2 | A | G | G | 0.1332 | 0.1096 | 0.158 | 0.5909 |
| 3 | A | A | G | 0.1309 | 0.1543 | 0.1073 | 0.7217 |
| 4 | G | A | G | 0.119 | 0.1665 | 0.0707 | 0.8407 |
| 5 | A | A | A | 0.0745 | 0.0646 | 0.0855 | 0.9152 |
| 6 | G | A | A | 0.0547 | 0.0603 | 0.049 | 0.9699 |
| 7 | G | G | A | 0.0218 | 0.0243 | 0.0207 | 0.9917 |
| 8 | G | G | G | 0.0083 | 0.0098 | 0.0064 | 1 |

Snp1: rs3850641; Snp2: rs1234313; Snp3: rs10912580

**Supplementary Table 5** Haplotype and sex cross-classification interaction table (n=368, crude analysis)

|  |  | **female** | **male** |
| --- | --- | --- | --- |
| **Haplotype** | **Frequency** | **OR (95% CI)** | **OR (95% CI)** |
| **AGA**** | 0.4578 | 1.00 | **0.31 (0.11 - 0.87)** |
| **AGG** | 0.1332 | 0.71 (0.40 - 1.27) | 1.18 (0.37 - 3.74) |
| **AAG** | 0.1304 | **0.36 (0.20 - 0.66)** | **0.32 (0.13 - 0.83)** |
| **GAG** | 0.1197 | **0.27 (0.13 - 0.55)** | **0.03 (0.00 - 0.26)** |
| **AAA** | 0.0747 | 0.75 (0.35 - 1.63) | 0.44 (0.16 - 1.19) |
| **GAA** | 0.0543 | 0.45 (0.20 - 1.01) | 2.81 (0.22 - 35.35) |
| **GGA** | 0.0219 | 0.47 (0.12 - 1.96) | 0.79 (0.07 - 8.75) |
| **rare** | 0.0079 | 0.28 (0.01 - 5.16) | Inf |
| **Interaction p-value: 0.041** | | | |

** Alleles are related to rs3850641, rs1234313 and rs10912580 polymorphisms, respectively in all the reported haplotypes.
